# Intercultural doctor-patient communication in daily outpatient care: relevant communication skills

**DOI:** 10.1007/s40037-016-0288-y

**Published:** 2016-09-16

**Authors:** Emma Paternotte, Fedde Scheele, Conny M. Seeleman, Lindsay Bank, Albert J. J. A. Scherpbier, Sandra van Dulmen

**Affiliations:** 1 Department of Healthcare Education, OLVG Hospital, Amsterdam, The Netherlands; 2 Medical School of Sciences, Vu University Medical Centre, Amsterdam, The Netherlands; 3 Department of Social Medicine, University Medical Centre, Utrecht, The Netherlands; 4 Institute for Medical Education, Faculty of Health, Medicine and Life Sciences, Maastricht University, Maastricht, The Netherlands; 5 NIVEL (Netherlands Institute for health services research), Utrecht, The Netherlands; 6 Department of Primary and Community Care, Radboud University Medical Centre, NIjmegen, The Netherlands; 7 Faculty of Health Sciences, University College of Southeast Norway, Drammen, Norway

**Keywords:** Doctor-patient communication, Intercultural communication, Communication skills, Clinical practice, Reflective practice, Communication behaviour, Medical education

## Abstract

**Introduction:**

Intercultural communication (ICC) between doctors and patients is often associated with misunderstandings and dissatisfaction. To develop ICC-specific medical education, it is important to find out which ICC skills medical specialists currently apply in daily clinical consultations.

**Methods:**

Doctor-patient consultations of Dutch doctors with non-Dutch patients were videotaped in a multi-ethnic hospital in the Netherlands. The consultations were analyzed using the validated MAAS-Global assessment list in combination with factors influencing ICC, as described in the literature.

**Results:**

In total, 39 videotaped consultations were analyzed. The doctors proved to be capable of practising many communication skills, such as listening and empathic communication behaviour. Other skills were not practised, such as being culturally aware and checking the patient’s language ability.

**Conclusion:**

We showed that doctors did practice some but not all the relevant ICC skills and that the ICC style of the doctors was mainly biomedically centred. Furthermore, we discussed the possible overlap between intercultural and patient-centred communication. Implications for practice could be to implement the relevant ICC skills in the existing communication training or develop a communication training with a patient-centred approach including ICC skills.

## What this paper adds

- Intercultural communication is challenging for doctors.
- It is however sparsely trained.
- Many intercultural communication skills are known.
- Unknown is, however, what intercultural communication skills medical specialists apply and what should be trained more.
- Implications of the study are the possible overlap between patient-centred communication and intercultural communication.
- This could facilitate development and implementation of intercultural communication skills in medical education.

## Introduction

Effective, patient-centred communication between doctors and patients is essential for delivering high-quality patient care [1]. Competent communication by doctors improves health outcomes, enhances patient satisfaction, and contributes to doctors’ job satisfaction [2]. In the context of a multicultural society, however, effective communication could be hindered by cultural differences between the doctor and the patient [3]. Intercultural communication (ICC), which in this article is defined as communication between a doctor of the dominant ethnic origin and an ethnic minority patient, potentially causes misunderstanding and reduces interpersonal interactions, which may lead to a lower quality of care [4]. Napier et al. stated that ‘the systematic neglect of culture in health and healthcare is the single biggest barrier to the advancement of the highest standard of health worldwide’ [5].

The theoretical fundaments of ICC between doctors and patients have gained attention in the last few years [6–8]. In a recent review, a conceptual framework of influencing factors in ICC is presented. This framework is constructed based on 145 included articles with a variety of evidence about ICC between the doctor and the patient. The factors influencing ICC, such as the role of the family in a conversation, the doctor’s awareness of the effects of differences in ethnic background, or the patient’s expectations of a conversation with the doctor, were translated into communication skills. These skills are of great importance in daily clinical practice and hence should be implemented in medical education [6].

The importance of the use of certain communication skills depends on the relevance of that skill in the specific context [9, 10]. In general, however, professional communication requires adaptation to the specific characteristics of the patient and the situation. Therefore, different contexts, such as differences in ethnic origin between the doctor and the patient, should be explicitly addressed [3, 6, 7, 11, 12].

While the theoretical knowledge on ICC skills and the necessity of using these skills have been established [6, 8, 13], several researchers argue that the scientific field of ICC between doctors and patients in real practice is still too small to develop focused training in ICC [7, 14] and that just giving feedback does not cover the full picture of skilled medical communication [15]. It is, for example, unknown which ICC skills are already used by doctors and how they are used. To develop knowledge about the ICC skills used, and therefore also the skills that they do not practice properly, there is a need to further explore which of these communication skills are applicable in the clinical setting, and which of them are already being practised [3, 13].

In this paper, we identify which ICC skills medical specialists use in real practice during those moments in the medical visit in which such skills are judged to be relevant. We addressed the following research question: Which ICC-influencing factors described in literature are recognizable in doctors’ communication skills in real practice?

## Methods

### Study design

In this observational study, doctor-patient consultations with ethnic minority patients and doctors of the Dutch ethnicity were videotaped and analyzed. The analysis focused on the doctor’s way of communicating and concentrated specifically on whether the ICC skills identified in a recent realist review were applied in daily practice [6].

## Setting and participants

Between September and December 2014, we videotaped conversations of gynaecologists, internists, urologists and orthopaedic surgeons in the outpatient clinics of the Sint Lucas Andreas Hospital in Amsterdam, the Netherlands. This district teaching hospital serves an urban multi-ethnic area. All Dutch medical specialists of the above-mentioned specialties of the Sint Lucas Andreas Hospital were asked to participate. Medical specialists were chosen since they are role models for residents and more experienced in communication with patients. The patients with a non-Dutch origin were included if they had an appointment for a new episode and the patient had not been seen by this doctor for a year or more. These patients could be of any origin and were not a *priori* selected. They were all referred by a general practitioner. The patients were asked about their place of birth and that of their parents before they were asked to participate. Informed consent of both the doctor and the patient was requested by the first author who then, if informed consent was obtained, installed the camera and left the room. Exclusion criteria were the presence of an interpreter, a patient with Dutch ethnicity, a doctor of non-Dutch origin, a follow-up consultation or a consultation that was partly done by somebody else, for example a medical student.

## MAAS-Global InterCultural Communication

Since a validated observation list for ICC did not exist, we combined the MAAS-Global, a validated instrument for assessing patient-centred communication [2, 16, 17], with the factors influencing ICC as found in a recent review [6]. Validation of any measure is a permanent process [18]. The strong base of the items scored in theory supports the content validity of the measure. Since the MAAS-Global was developed in the Netherlands to assess communication skills of general practitioner residents in real practice, we considered it a suitable instrument to use for the observation of communication skills of doctors in the hospital [19]. The MAAS-Global has been used in research settings as well [20]. Combining the two protocols, i. e. the factors influencing ICC and the MAAS-Global, was possible because there is an overlap between the categories of the MAAS-Global and those used to classify the influencing factors in the review. The combination of the MAAS-Global and the ICC-influencing factors provided a framework for coding ICC skills, which could then be observed. The resulting observational scale, the MAAS-Global ICC (Appendix), includes 52 communication skills to be analyzed on a dichotomous scale as ‘present or absent’ and a 4-point Likert scale to indicate the relevance of each skill for the consultation under observation. This observation and analysing is done per section of the communication, e. g. opening or exploration of reason for encounter (Appendix). In the results section, we report on the relevant skills which were present or absent. Because the MAAS-Global ICC is an extensive list, containing many items, the result section includes the communication skills of the MAAS-Global ICC that were found to be relevant as absent or present in at least 40 % of the consultations.

## Measures and analysis

The adapted MAAS-Global ICC was tested on face validity within the project team, which consisted of specialists from different fields of expertise (medical, cultural competence, communication in healthcare, medical education). The first author (EP) observed and analyzed all the included videotaped consultations. The videotaped consultations were also independently observed and analyzed by one of four second observers (CS, LB, LR, TA), who all watched 9–10 videotaped consultations each. After the first independently observed consultation, the intraclass correlation coefficient (Cohen’s kappa) was calculated and discussed between EP and each second observer. Thereafter, EP and the second observer independently scored three consultations, and once again the intraclass correlation coefficient was calculated. If the Cohen’s kappa was below 0.6, the videotaped consultation and scoring were discussed to check if the observers could reach a higher level of agreement. Before discussion, the mean Cohen’s kappa between the observers ranged from 0.47 to 0.59. After a discussion between the observers, the mean Cohen’s kappa ranged from 0.67 to 0.82. Scoring of the videotaped consultations was analyzed with SPSS 21.

After each consultation, doctors were asked if they were satisfied about the consultation. Also, doctors had to write down if they had enough time for the consultation.

## Results

In total, 18 doctors were asked to participate and 17 doctors agreed to participate. One doctor refused because he found it difficult to ask his oncology patients. Of these 17 doctors, 69 consecutive patients of non-Dutch origin were asked to participate. Of these patients, 41 gave informed consent. The other 28 patients refused to participate, mostly because of privacy reasons. Two of the 41 videotaped consultations were excluded, one because the doctor was of non-Dutch origin and the other one because the videotape lacked sound.

Table 1 shows the characteristics of the 39 included videotaped consultations. Furthermore, Table 2 presents the relevant communications skills demonstrated by the doctors. Table 3 lists the communication skills that were not used by the doctors but that the observers considered to be relevant in the specific context of an intercultural conversation. After the consultation, all doctors noted that they were satisfied with the consultation and that they thought they had enough time for the consultation.

**Table 1:** Characteristics of the videotaped consultations

	Number of consultations (*n* = 39)	Ethnicity (non-Western^a^/Western^b^) *n* = 39	Gender (M/F)	Mean age (y)	Mean years of practice as medical specialist (y)	Mean length videos (min)
Patients included (%)	–	32/7 (85/15)	21/18 (54/46)	46.3	–	–
Specialty of the doctor^c^						
Gynaecology & Obstetrics	7	–	2/3	46.0	12.4	17.4
Internal medicine	15	–	5/1	44.3	16.0	14.6
Urology	5	–	3/0	57.7	21.0	7.8
Orthopaedics	12	–	4/0	52.5	15.8	13.0

^a^Afghanistan, Turkey, Morocco, Surinam, Nicaragua, Nepal, Nigeria, Cuba, Pakistan, China

^b^Poland, Great Britain, Germany, Belgium, Australia, Hungary

^c^Doctors were all of Dutch origin

**Table 2:** An overview of skills, present in at least 40 % of the consultations, that the doctors used: present communication skills

Present communication skills^a^ The doctor …
Listens
Demonstrates reliability (being friendly and having an open attitude)
Makes appointments: who, what, when
Takes the time
Has an unprejudiced attitude
Shows empathic behaviour
Applies an adequate time schedule
Gives concrete explanations
Shows respect for the patient
Uses concrete language
Explains referral to other healthcare workers
Listens actively
Shows concern, is inviting and sincere, commiserates by means of eye contact and non-verbal behaviour, shows compassion for the patient
Commiserates with verbal reactions
Has an open attitude (shows possibilities verbal/non-verbal to give the patient space for their story)
Responds to non-verbal behaviour and keywords
Gives information in small amounts
Tries to empathize with the patient’s emotions
Explains cause and relation of the complaint within the context of the expectations of the patient
Reflects on the feelings of the patient
Uses different ways to give explanations
Announces stages of the conversation
Treats the patient with care and respect during physical examination
Checks if the patient and/or relatives understand the explanation

^a^The skills in the table are presented from most to less present

**Table 3:** An overview of skills, absent in at least 40 % of the consultations, that the doctors did not use but that were relevant within the context of these consultations: absent communication skills

Absent communication skills^a^ The doctor did not …
Check expectations regarding the consultation/healthcare
Ask about the patient’s feelings
Ask about the relatives’ emotions
Show awareness of his own cultural and professional context
Check foreknowledge of the patient about diagnosis or expected policy
Summarize the patient’s story
Explore the reason for the consultation, wishes and expectations
Explore reaction of information transfer to the patient’s context
Demonstrate being alert to possible cultural aspects when asking for the reason for the consultation
Show awareness of cultural differences
Show to have learned from previous consultations with ethnic minority patients
Ask if the patient understood the information
Check if the patient and/or family understood the explanation
Adapt cultural differences in diagnosis and policy
Observe cultural differences
Check the language ability of the patient
React adequately to possible cultural differences

^a^The skills in the table are presented from most to less absent

### Observed communication skills

Doctors showed a variety of ICC skills that facilitated the communication. For example, in most consultations doctors adequately employed concrete language, listening and empathic behaviour toward the patients, such as reflecting the patient’s feelings and demonstrating concern. Also, doctors gave concrete explanations, for example using drawings to explain an X‑ray. Most conversations were not hurried, and most doctors had an adequate time schedule. All these present skills were considered relevant by the observers because, in this way respect, reliability and an unprejudiced attitude were shown.

In many consultations the doctors used a biomedical style of communication, in which they focused on their own agenda with biomedically structured questions and fewer possibilities for the patient to give input.

### Absent communication skills

ICC language skills include checking the patient’s language ability, which was absent in 17 consultations where it would have been relevant to do. In 37 consultations the main language spoken was Dutch. In two it was English. Absent ICC skills, such as awareness of cultural differences, e. g. the doctor says something about treatment habits in the Netherlands or asks the patient about his/her cultural habits for the specific disease, and adaptation of the diagnosis and treatment policy to the context of the patient, e. g. the doctor asks if the prescription use of the medication is possible and satisfactory for the patient, were considered relevant because these skills facilitate mutual understanding and respect.

These ICC skills were sometimes difficult to score, because they were elusive and not explicit. For example, the doctor did not always directly address a patient’s cultural background, but tried to get insight into the patient’s perspective by figuring out what the patient thought to be the cause of the complaint (e. g. pain). Also, many doctors did not check the foreknowledge of the patient about the diagnosis and treatment policy. The relevance of attention to cultural differences was emphasized in the doctor’s explicit communication. For example, doctors did not take the patient’s context into account when proposing a policy, such as medication intake or dietary advice, and they had difficulties shifting from their biomedical communication style to the context and expectations of the patient. When the conversation was mainly biomedical, it was difficult to determine if the doctors were aware of their own cultural and professional context. In a few conversations the doctors mentioned their own cultural origin, for example by explaining how a treatment is carried out in the Netherlands. This, however, did not linearly cause doctors to pay attention to cultural differences. Summaries were not often used in the conversation, although this could have structured the conversation and it could have helped both the doctor and the patient to check if specific information was understood correctly.

Other skills that were absent but relevant lay in the field of expectation management, showing interest in the patient’s family and checking if the patient understood the information given by the doctor, which was relevant as it might have helped to clarify possible misunderstandings. An example of expectation management is exploring the patient’s view on the reason for the consultation or the patient’s expectation of the consultation. However, if doctors used questions aimed at clarifying the patient’s expectations, which was not done in 62 % but used in 38 % of the consultations, this proved to facilitate ICC and direct the communication into a more patient-centred approach, depending on the way they were phrased. For example, after listening to a complex account of the patient’s complaints, one doctor asked: ’What do you expect from me? Would you like me to reduce the pain, or is it something else?’

## Discussion

In this observational study, we focussed on relevant ICC skills of medical specialists in real practice. The medical specialists in this study proved to be capable of practising many communication skills, such as listening, showing empathic communication behaviour and being open and respectful to the patient. Other skills were not practised although they were relevant in the intercultural context, such as being culturally aware, checking the patient’s language ability, checking if the patient understood and exploring the reason for the consultation. The communication style of the doctors was often a biomedical style.

The use of a biomedical style in these intercultural conversations is surprising, since ICC requires a patient-centred focus with specific attention to the patient’s bio-psychosocial needs, because of the vulnerability for misunderstandings of ethnic minority patients [13, 21]. Our study showed that the doctors did not properly apply a number of specific ICC skills, such as adapting diagnosis and treatment policy to the cultural context. However, they also did not practice certain generic communication skills, such as exploring the reason for the consultation or checking the feelings of the patient, which is striking because we included medical specialists who could be expected to have learned how to practice these communication skills in their undergraduate and postgraduate training This is a valuable finding, as medical specialists function as role models for postgraduate trainees [22], which is, however, not always true due to the transfer problems of communication skills from training to trainee [23]. In all medical specialty training programmes in the Netherlands there is some formal communication training. However, this usually takes place during practice and not as additional training. The communication training is most extensive for residents of the training to become a general practitioner [23].

Nowadays, doctors in Western countries are taught to use a patient-centred communication style [8, 13, 21, 24]. Patient-centred communication has similarities with ICC, such as the responsibility of the doctor for the non-medical or interpersonal aspects of the communication [25]. The interpersonal aspects of care, for example trust, respect and empathy, are key determinants of patient satisfaction [13, 21]. As was mentioned above, we found missing generic communication skills, such as exploring the reason for the consultation, checking if the patient understood, and expectation management. These are skills of patient-centred communication as well [21]. In an intercultural context, patient-centred communication is probably even more important, because the balance in the interpersonal aspects of the communication is harder to find when doctor and patient have different norms and values. Intercultural and patient-centred communication have not been formally integrated together in medical education, although the function of both intercultural and patient-centred communication is to improve healthcare quality in similar ways and the used skills for patient-centred communication and ICC show similarities. Therefore, patient-centred communication and ICC should be incorporated in medical education, so that doctors will not have to learn two different approaches [13].

It was striking that the doctors all said they were satisfied with their conversation, while the observers concluded that doctors did not practice the relevant ICC skills. An explanation could be that the doctors need to be confronted with their communication behaviour before they can improve their communication skills [15]. Finally, we need to say that the complexity of ICC cannot be grasped in a list of do’s and don’ts. It is not a matter of learning only one skill for ICC but of learning a complete set of skills and being able to apply these in the right way at the right time. It is the complete set of behaviours which makes a doctor a good intercultural communicator, and communication training is not a ‘one size fits all’ training [5].

## Conclusion

We showed that doctors did practice some but not all the relevant ICC skills and that the ICC style of the doctors was mainly biomedically centred. Hence, it is unlikely that postgraduate medical trainees will acquire all the required ICC skills merely by modelling their behaviour on the example of their clinical supervisors. Furthermore, we discussed the possible overlap between intercultural and patient-centred communication. This overlap and the absence of skills in both these domains suggest that integrating patient-centred communication and ICC training may contribute substantially to the development of medical education for postgraduates and medical specialists.

## Strengths and limitations

This observational study provided the opportunity to examine the application of ICC skills in real practice. A strength of this study was the focus on specialists instead of trainees, because medical specialists function as role models for postgraduate trainees. Another strength was that the consultations were videotaped before they were analyzed, and that the tapes were analyzed by observers from different areas of expertise, so that the data could be viewed from several perspectives. Since the groups of medical specialists were small, it was not possible to further analyze the influence of age and years of experience. Besides, the study population was too small to assess differences in communication styles between the doctors.

Another limitation was that we did not ask the patients for their years of residence in the Netherlands. The exact influence of this on ICC is hard to determine. On one hand, being in the Netherlands for a longer period could positively suggest an influence of the ICC with a native doctor. On the contrary, many people continue to identify themselves with their country of origin [26], which could suggest that years of residence in the Netherlands is less important.

## Implications for medical education

Based on the results of our observational study of daily outpatient care and the points mentioned in the discussion, we would advise to extend the already existing communication training for postgraduate medical education with ICC-specific skills, such as asking about the language proficiency of patients or checking if the proposed treatment plan fits into the cultural habits of the patient. Elaborating ICC training could include discussion of doctors’ own video consultations with peers in the presence of a communication expert. Besides, we would advise that medical specialists also embrace the concept of lifelong learning and that they attend communication training focused on patient-centred communication that includes ICC.

## Future research

Many of the elements of the MAAS-Global ICC that we used seemed to be relevant for communication with every patient. Future research could study whether this is true, and should further explore the overlap between ICC and patient-centred communication. In the present study, we focused on the doctor from the third person, the observer. Also, it appears to be important to evaluate doctors’ needs for ICC skills and patients’ preferences and satisfaction with their doctors’ communication skills. Further research could be a reflective practice study with doctors based on their videotaped consultations. Another further research possibility is to compare the patient-centred communication skills and ICC skills of doctors. This could facilitate the development of training focused on relevant communication skills.

## Appendix

**Table 4:** MAAS-Global ICC observation scale

The doctor …
*Opening*
Checks the language ability of the patient
Checks who is the formal speaker of the family
Asks to the relatives for their connection with the patient
Listens
Reacts adequately to possible cultural differences
*Reason For Encouter*
Demonstrates being alert to possible cultural aspects when asking for the reason for the consultation
Checks reasons of encounter of the relatives
Checks expectations regarding the consultation/healthcare
*Physical Examination*
Treats the patient with care and respect
*Diagnosis*
Explains cause and relation of the complaint within the context of the expectations of the patient
Checks if the patient and/or relatives understood the explanation
*Policy*
Adapt cultural differences in diagnosis and policy
Checks with the relatives if they understand the choice of policy
Makes appointments: who, what, when
Explains referral to other healthcare workers
*Explore*
Explores the reason for consultation, wishes and expectations
Explores the perception of the relatives
Recognizes misunderstanding caused by a language barrier
Explores the reaction of information transfer to the patient’s context
Responds to non-verbal behaviour and keywords
Responds to cues/key words which are related to cultural differences
*Emotions*
Asks about the patient’s feelings
Reflects on the feelings of the patient
Asks about the relatives’ emotions
Listens actively
Tries to empathize the patient’s emotions
*Information Transfer*
Checks the foreknowledge of the patient about diagnosis or expected policy
Gives information in small amounts
Gives concrete explanations
Uses concrete language
Asks if the patient understood the information
Uses different ways to give explanations
Pays attention to pronunciation
Uses attributes for explanation
*Summarize*
Summarizes the patient’s story
Summarizes in his own words, concise
Attempts
*Structure*
Applies an adequate time schedule
Takes the time
Announces stages of the conversation
*Empathy*
Shows concern, is inviting and sincere, commiserates by means of eye contact and non verbal behavior, shows compassion for the patient
Commiserates with verbal reactions
Observes cultural differences
Shows empathic behavior
Has an open attitude
Shows respect for the patient
*Consult Evaluation*
Has an unprejudiced attitude
Demonstrates reliability
Shows awareness of his or her own cultural and professional context
Shows awareness of cultural differences
Speaks more languages of words of another language
Shows to have learned from previous consultations with ethnic minority patients

## References

[1] Teutsch C (2003). Patient-doctor communication. Med Clin North Am.

[2] Schirmer JM, Mauksch L, Lang F (2005). Assessing communication competence: A review of current tools. Fam Med.

[3] Betancourt JR, Green AR, Carrillo JE, Park ER (2005). Cultural competence and health care disparities: Key perspectives and trends. Health Aff (Millwood).

[4] Ferguson WJ, Candib LM (2002). Culture, language, and the doctor-patient relationship. Fam Med.

[5] Napier AD, Ancarno C, Butler B (2014). Culture and health. Lancet.

[6] Paternotte E, van Dulmen S, van der Lee N, Scherpbier AJJA, Scheele F. Factors influencing intercultural doctor-patient communication: A realist review. Patient Educ Couns. 2014; doi:10.1016/j.pec.2014.11.018. 10.1016/j.pec.2014.11.018 25535014

[7] Schouten BC, Meeuwesen L (2006). Cultural differences in medical communication: a review of the literature. Patient Educ Couns.

[8] Teal CR, Street RL (2009). Critical elements of culturally competent communication in the medical encounter: A review and model. Soc Sci Med.

[9] Essers G, van Dulmen S, van Weel C, van der Vleuten C, Kramer A (2011). Identifying context factors explaining physician’s low performance in communication assessment: An explorative study in general practice. BMC Fam Pract.

[10] Essers G, Dielissen P, van Weel C, van der Vleuten C, van Dulmen S, Kramer A (2015). How do trained raters take context factors into account when assessing GP trainee communication performance? An exploratory, qualitative study. Adv Health Sci Educ Theory Pract.

[11] Essers G, van Dulmen S, van Es J, van Weel C, van der Vleuten C, Kramer A (2013). Context factors in consultations of general practitioner trainees and their impact on communication assessment in the authentic setting. Patient Educ Couns.

[12] Roberts C, Moss B, Wass V, Sarangi S, Jones R (2005). Misunderstandings: A qualitative study of primary care consultations in multilingual settings, and educational implications. Med Educ.

[13] Saha S, Beach MC, Cooper LA (2008). Patient centeredness, cultural competence and healthcare quality. J Natl Med Assoc.

[14] Saha S, Beach MC (2011). The impact of patient-centered communication on patients’ decision making and evaluations of physicians: A randomized study using video vignettes. Patient Educ Couns.

[15] van den Eertwegh V, van der Vleuten C, Stalmeijer R, van Dalen J, Scherpbier A, van Dulmen S (2015). Exploring residents’ communication learning process in the workplace: A five-phase model. PLoS ONE.

[16] Ram P, Grol R, Rethans JJ, Schouten B, van der Vleuten CP, Kester A (1999). Assessment of general practitioners by video observation of communicative and medical performance in daily practice: issues of validity, reliability and feasibility. Med Educ.

[17] Ram P, van Thiel J, van Dalen J (2003). MAAS-Global Manual 2000.

[18] Streiner DL, Norman GR (2008). Health measurements scales: A practical guide to their development and use.

[19] Veldhuijzen W, Ram P, van der Weijden T, Wassink M, van der Vleuten C (2007). Much variety and little evidence: a description of guidelines for doctor-patient communication. Med Educ.

[20] Hobma S, Ram P, Muijtjens A, van der Vleuten C, Grol R (2006). Effective improvement of doctor-patient communication: a randomised controlled trial. Br J Gen Pract.

[21] Mead N, Bower P (2000). Patient-centredness: A conceptual framework and review of the empirical literature. Soc Sci Med.

[22] Watling C, Driessen E, van der Vleuten CP, Lingard L (2012). The Accreditations Council for Graduate Medical Education (ACGME). Med Educ.

[23] van den Eertwegh V, van Dulmen S, van Dalen J, Scherpbier AJ, van der Vleuten CP (2013). Learning in context: Identifying gaps in research on the transfer of medical communication skills to the clinical workplace. Patient Educ Couns.

[24] Ball JW, Dains JE, Flynn JA, Solomon BS, Stewart RW, Mosby (2014). Cultural competency. Seidel’s guide to physical examination.

[25] Grol R, de Maeseneer J, Whitfield M, Mokkink H (1990). Disease-centred versus patient-centred attitudes: comparison of general practitioners in belgium, britain and the netherlands. Fam Pract.

[26] Hofstede G, Hofstede GJ (2005). Allemaal anders denkenden. Omgaan met cultuurverschillen.

